# Metabolic Basis of Cognitive Improvement Associated With Active B Vitamin Supplementation in Cognitively Impaired Elderly Subjects – A Metabolomics Study

**DOI:** 10.3389/fmed.2022.864152

**Published:** 2022-04-27

**Authors:** Haiming Zhou, Yuanyuan Wu, Binhua Jiang, Bowen Li, Martin Li, He Tian, Guanghou Shui, Sin Man Lam, Timothy Kwok

**Affiliations:** ^1^LipidALL Technologies Company Limited, Changzhou, China; ^2^Health Management Center, The Second Affiliated Hospital of Chongqing Medical University, Chongqing, China; ^3^Department of Medicine and Therapeutics, Prince of Wales Hospital, The Chinese University of Hong Kong, Shatin, Hong Kong SAR, China; ^4^State Key Laboratory of Molecular Developmental Biology, Institute of Genetics and Developmental Biology, Beijing, China

**Keywords:** metabolomics, B vitamins, aspirin, cognitive impairment, dementia

## Abstract

Intervention studies with active B vitamin supplementation in cognitively impaired individuals have yielded varying results in randomized controlled trials. In addition, a negative interaction of active B vitamin supplementation with aspirin usage on cognitive outcome was noted, but the molecular basis of the interaction has largely remained unknown. To investigate the metabolic basis of cognitive improvement brought about by active B vitamin supplementation, we conducted an extensive metabolomics analysis covering 302 identified metabolites on the baseline and 24-month serum samples from a cohort of 137 subjects randomly assigned to active supplementation or placebo. Pathway analysis uncovered enhanced gluconeogenesis and War-burg effects underlying cognitive improvement in non-aspirin users supplemented with active B vitamins. In addition, metabolomics revealed that aspirin usage may interact with B vitamin supplementation by altering gut microbial metabolism, particularly in terms of propionate production. Lastly, our omics data suggest that varying capacities to assimilate B vitamins at baseline, possibly mediated by differences in gut microbial composition, may underlie variations in inter-individual responses to active B vitamin supplementation.

## Introduction

Approximately 50 million people across the globe live with dementia. The number is projected to increase to 152 million by the year 2050, especially in low-income and middle-income countries where close to two-thirds of the people afflicted with dementia reside (1). Dementia encumbers daily activities and financially strains the public health sector and the economy, with global costs estimated at USD one trillion annually (1). While the incidence of dementia is strongly correlated with age, dementia does not constitute normative aging and is a true disease instigated by exposure to genetic and environmental risk factors. Elevated homocysteine and lower-than-normal concentrations of B vitamins, which include folate, vitamin B12 and vitamin B6, denote candidate risk factors for both Alzheimer’s disease and vascular dementia (2). A plethora of cross-sectional and longitudinal studies comprising more than 36,000 subjects has demonstrated associations between cognitive impairment or dementia with homocysteine and/or B vitamins. Biologically plausible mechanisms for the beneficial action of B vitamins toward cognition have been proposed, but details await to be settled (2–4). Intervention studies with B vitamins in cognitively impaired individuals, however, have not yielded consistently positive results (5–9). Recent randomized trials uncovered a significant interaction between B vitamins and aspirin usage on cognitive function; and that B vitamin supplementation was associated with significantly favorable effects toward global cognitive functioning and whole brain atrophy rate in older people with mild cognitive impairment (MCI) who were not taking aspirin, but not in those who took aspirins concurrently (10, 11). Given these observations, it is imperative for research to delve into the molecular aspects of B vitamins and aspirin interaction that may underlie the clinical outcome of B vitamin supplementation in elderly subjects. High-coverage metabolomics offers an unbiased, inclusive approach to dissect molecular alterations in response to treatment intervention (12), and can unveil potential molecular pathways that underlie observed phenotypes (13).

Herein, we conduct an extensive metabolomics profiling that comprises 302 identified metabolites on pre-intervention (i.e., baseline) and post-intervention serum samples from 137 subjects randomly assigned to two main treatment interventions over a course of 24 months, which included active ingredient (folic acid and Vitamin B12) supplementation and placebo. Aspirin usage was monitored in these subjects, which further segregated the treatment groups into active + non-aspirin and active + aspirin, and their corresponding control groups being placebo + non-aspirin and placebo + aspirin, respectively. At the end of the two-year treatment intervention, cognitive outcome in these subjects were measured by the clinical dementia rating scale sum of boxes scores (CDR-SOB) (14). Differences in metabolomics profiles and dysregulated metabolic pathways between the treatment and control groups at the end of two-year intervention were examined in consideration of measured outcome i.e., CDR-SOB to uncover metabolic pathways implicated in the different treatment interventions and their associated cognitive outcomes.

## Materials and Methods

### Study Participants

Patients aged ≥ 65 years were screened by the Montreal cognitive assessment (MoCA) test in the specialist medical outpatient clinics at the Prince of Wales Hospital (PWH) during April 2013 and July 2016. After obtaining written informed consent, subjects who had a MoCA score lower than 22, which suggested MCI, had fasting blood taken for serum homocysteine analysis. Those with homocysteine ≥ 10 μmol/L were further assessed of co-existing illnesses and a neurological examination, to exclude patients with dementia or clinical depression, or those with peripheral neuropathy, renal failure, anemia, disabling stroke and those who were receiving B vitamin supplementation or centrally acting medications. In total, 279 out of 975 outpatients were enrolled in this clinical trial (10). The trial was approved by the Medical Ethics Committee of Chinese University of Hong Kong and Hospital Authority of Hong Kong (CUHK_CCT00373).

### Clinical Design

**FIGURE 1:**
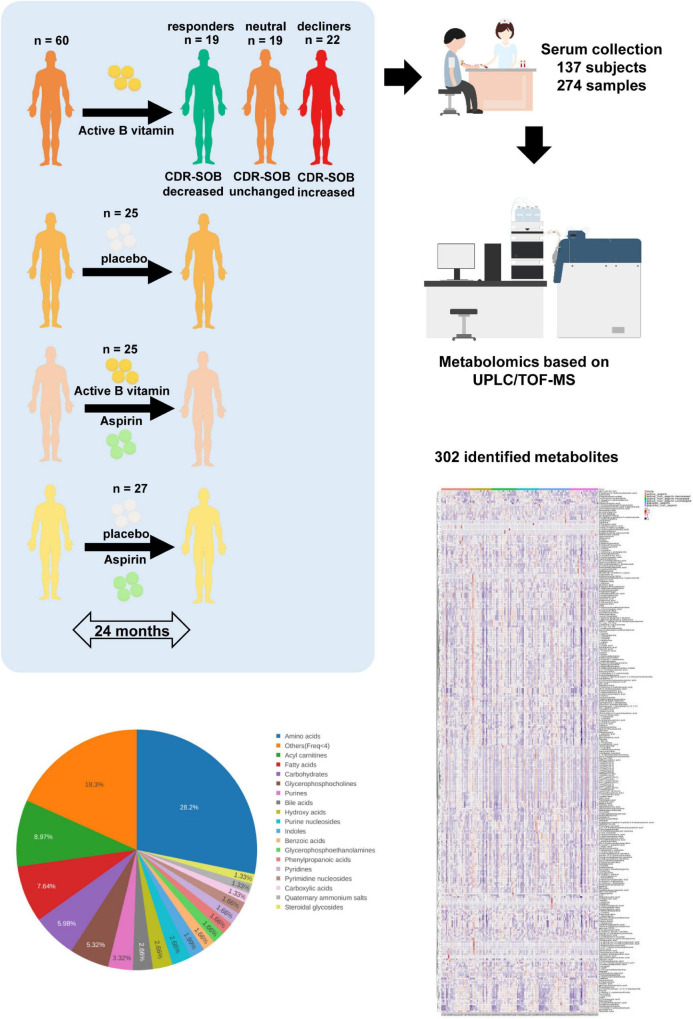
Schematic diagram of clinical study design. Subjects were randomly assigned to active B vitamin supplementation or placebo. Aspirin usage was monitored, which further segregated the subjects into four clinical groups. To examine the molecular basis of differing responses to active B vitamin supplementation, subjects under active B vitamins, non-aspirin group were further segregated into three groups based on cognitive outcome measured by 24-month changes in CDR-SOB. Baseline and 24-month serum samples were analyzed using untargeted metabolomics covering more than 300 identified metabolites.

Subjects aged 65 years or more in the specialist medical outpatient clinics at the Prince of Wales hospital between April 2013 and July 2016 were screened for cognitive impairment. After obtaining written informed consent, fasting blood was taken from individuals with MCI for serum homocysteine analysis, and subjects with elevated serum homocysteine (more than 10 μmol/L) were recruited into the study (see section “Study Participants” for more details). Subjects were randomly assigned into active ingredient intervention (400 μg/d of folic acid and 500 μg/d of vitamin B12) and placebo (two placebo tablets per day) groups, and their aspirin usage was recorded. As the effects of B vitamin supplementation toward cognitive outcome have been conflicting based on past literature (7, 9), we included a greater number of subjects into the active + non-aspirin group of our study to allow a comparison of metabolome profiles based on two-year cognitive outcome. At the end of the 24-month intervention, changes in CDR-SOB were used to further delineate subjects under the active + non-aspirin group into sub-categories based on cognitive outcome, which included those with longitudinal decrease in CDR-SOB (positive outcome) i.e., responders, no significant changes (neutral), and longitudinal increase in CDR-SOB (negative outcome) i.e., decliners (Figure 1 and Table 1).

**TABLE 1:** Clinical cohort summary.

	Active + aspirin	Active + non-aspirin, CDR-SOB increased	Active + non-aspirin, CDR-SOB unchanged	Active + non-aspirin, CDR-SOB decreased	Placebo + aspirin	Placebo + non-aspirin	*P*-value
	
*n*	25	22	19	19	27	25	
Age [mean (SD)]	76.5 (6.03)	76.9 (4.59)	75.0 (5.34)	76.8 (4.3)	77.96 (4.38)	78.0 (5.81)	0.4209
Change in CDR-SOB over 24 months [mean (SD)]	0.60 (1.46)	1.36 (1.15)*	0 (0)	−0.71 (0.35)*	−0.17 (0.67)	0.54 (0.095)*	*Indicates statistical significant changes compared to baseline
Sex							0.6676
Male	18 (72%)	16 (72.8%)	13 (68.4%)	11 (57%)	17 (63%)	16 (64%)	
Female	7 (28%)	6 (27.2%)	6 (31.6%%)	8 (42.1%)	10 (37%)	9 (36%)	

*Differences in age across groups were compared using Welch’s ANOVA. Changes in sex were compared using Chi-square’s test. Longitudinal changes in CDR-SOB were compared using paired t-test.*

### Serum Collection

The blood samples were taken after an overnight fasting for serum folate, vitamin B_12_, homocysteine and creatinine. The samples were temporarily stored in an icebox and transported within 2 h to the Department of Medicine and Therapeutics at PWH. The serum was then separated and kept under −80°C until further use.

### Metabolite Extraction

Polar metabolites were extracted from serum samples using a modified version of Bligh and Dyer’s protocol as described previously (15). Serums samples were incubated for 30 min at 1500 rpm and 4°C in extraction solvent containing chloroform: methanol (1:2) (v/v); and water was added at the end of the incubation to induce phase separation. Samples were then centrifuged for 10 min at 12000 rpm and 4°C. The upper aqueous phase containing polar metabolites was transferred into a clean 1.5 ml centrifuge tube, and dried using SpeedVac under aqueous mode. Dried extracts were resuspended in 2% acetonitrile in water for LC-MS analysis.

### Untargeted Metabolomics Analyses Based on UPLC-qTOF-MS

Metabolomics analysis was performed as previously described (12). The ACQUITY UPLC HSS T3 1.8 μm, 2.1 mm × 100 mm column (Waters, Dublin, Ireland) was used for reverse-phase chromatographic analysis, while the ACQUITY UPLC BEH Amide 1.7 μm, 2.1 mm × 100 mm column (Waters, Dublin, Ireland) was utilized for normal-phase chromatographic analysis. An Agilent 1290 II Ultra-performance Liquid Chromatographer (Agilent Technologies) coupled to 5600 Plus Quadrupole-time-of-flight MS (5600 Triple TOF Plus, SCIEX) was used to acquire the metabolome data. The MS parameters for detection were as follows, ESI source voltage positive ion mode +5.5 k V, negative ion mode −4.5 kV; vaporizer temperature, 500°C; drying gas (N2) pressure, 50 psi; nebulizer gas (N2) pressure, 50 psi; curtain gas (N2) pressure, 35 psi; The scan range was m/z 60–900. Information-dependent acquisition mode was used for MS/MS analyses of the metabolites. The collision energy was set at (±) 35 ± 15 eV. Data acquisition and processing were performed using Analyst^®^ TF 1.7.1 Software (Sciex, Concord, ON, Canada). All detected ions were extracted using MarkerView 1.3 (Sciex, Concord, ON, Canada) into Excel in the format of two dimensional matrix, including mass to charge ratio (m/z), retention time, and peak areas, and isotopic peaks were filtered. PeakView 2.2 (Sciex, Concord, ON, Canada) was applied to extract the MS/MS data, and spectral comparisons were performed with Metabolites database (Sciex, Concord, ON, Canada), HMDB, METLIN, and spectra acquired from standard reference compounds to annotate ion ID.

### Statistical Analyses

Metabolite identity, corresponding HMDB ID and class were assigned using an internal library. Metabolite abundance was measured by peak area. Hierarchical clustering was performed based on centered and scaled data with Euclidean distance and complete linkage, and was visualized using R package “ComplexHeatmap.” Average metabolite abundance was compared among all the groups using Kruskal–Wallis test, followed by *post hoc* Dunn’s test for pair-wise comparison. *P*-value less than 0.05 was considered as statistically significant. Metabolite set enrichment analysis (MSEA) was performed based on the metabolites with *P* < 0.05 over the small molecules pathway database (SMPDB) using R package “MetaboAnalystR.” The over-representation test for MSEA is hypergeometric test using all the annotated metabolites that can be mapped to HMDB IDs as the reference metabolome. The orthogonal partial least square discriminant analysis (OPLSDA) was performed using R package “ropls.”

## Results

### Differences in 24-Month Serum Metabolome Profiles Across Different Treatment Groups

**FIGURE 2:**
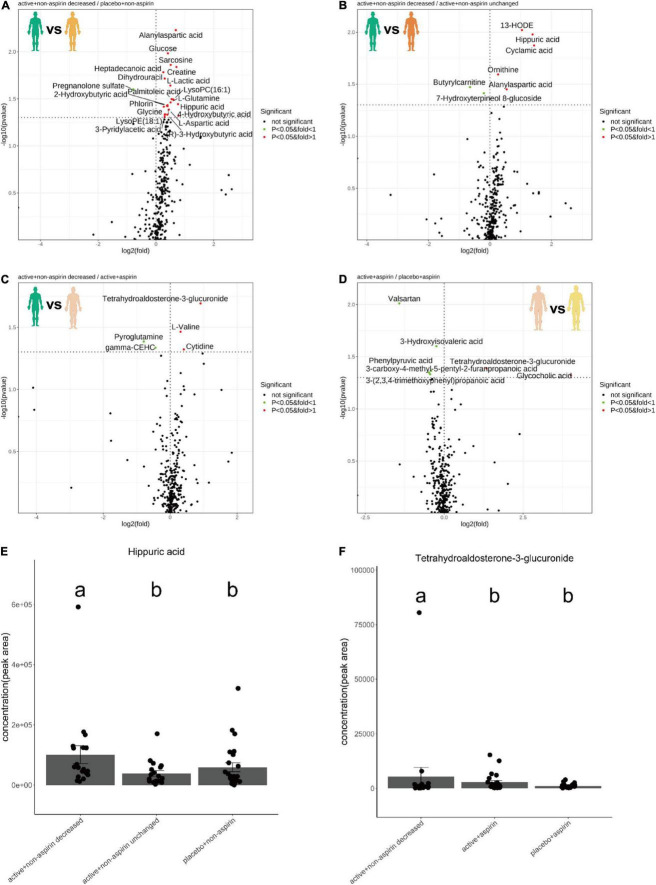
Volcano plots illustrate serum metabolite changes at 24-month in selected pairwise comparisons of clinical groups, which included **(A)** active B vitamin supplementation in non-aspirin users with reduction in CDR-SOB relative to non-aspirin users on placebo; **(B)** active B vitamin supplementation in non-aspirin users with reduction in CDR-SOB relative to those without changes in CDR-SOB; **(C)** active B vitamin supplementation in non-aspirin users with reduction in CDR-SOB relative to active B vitamin supplementation in aspirin users; **(D)** active B vitamin supplementation in aspirin users relative to aspirin users on placebo. Dotplots illustrate changes in the levels in **(E)** hippuric acid and **(F)** tetrahydro-aldosterone-3-glucuronide at 24 months. *P*-values from two-sided Dunn’s tests were displayed.

Comparing 24-month serum metabolomes of responders from the active + non-aspirin group relative to placebo + non-aspirin control group, the levels of metabolites including alanyl-aspartic acid, creatine, glucose, hippuric acid, L-glutamine, and L-lactic acid were increased, while that of pregnenolone sulfate was decreased (Figure 2A). Similarly, when compared to active + non-aspirin group with neutral cognitive outcome, i.e., unchanged CDR-SOB, serum level of hippuric acid was consistently higher in non-aspirin users who responded positively to active B vitamins supplementation (Figure 2B). Comparing the 24-month serum metabolome profiles of the active + aspirin group to responders from the active + non-aspirin group, the level of tetrahydro-aldosterone-3-glucuronide (THA-G) was lower in aspirin users (Figure 2C). THA-G was also lower in the placebo + aspirin control group compared to active + aspirin group (Figure 2D). Thus, active B vitamin supplementation increases serum level of THA-G, but concurrent aspirin usage reduces its level. Next, we utilized metabolic pathway analysis (MSEA) (16) to systematically investigate altered metabolic pathways between the treatment and control groups.

### Altered Metabolic Pathways Between Treatment and Control Groups

**FIGURE 3:**
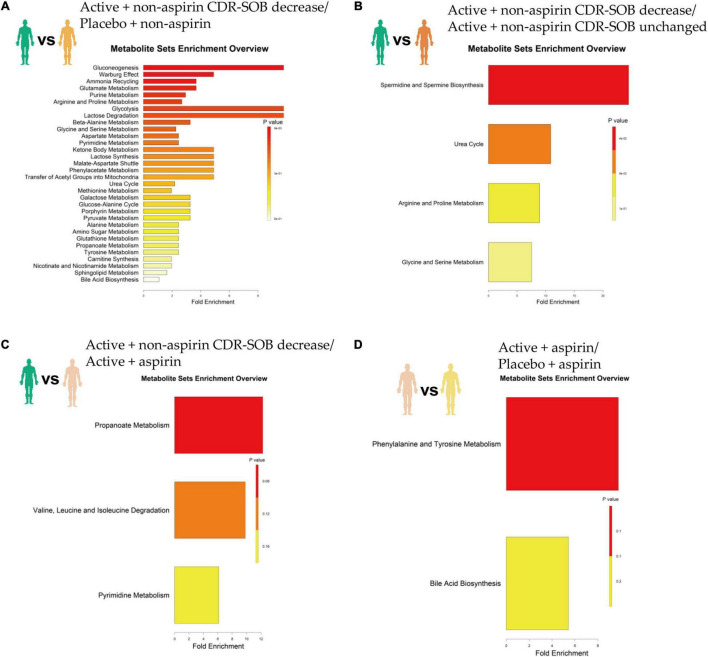
Top altered metabolic pathways based on metabolome changes at 24-month in selected pairwise comparisons of clinical groups, which included **(A)** active B vitamin supplementation in non-aspirin users with reduction in CDR-SOB relative to non-aspirin users on placebo; **(B)** active B vitamin supplementation in non-aspirin users with reduction in CDR-SOB relative to those without changes in CDR-SOB; **(C)** active B vitamin supplementation in non-aspirin users with reduction in CDR-SOB relative to active B vitamin supplementation in aspirin users; **(D)** active B vitamin supplementation in aspirin users relative to aspirin users on placebo.

Relative to placebo + non-aspirin group, active B vitamin supplementation in non-aspirin users who responded positively (i.e., longitudinal reduction in CDR-SOB) resulted in significantly elevated gluconeogenesis, enhanced Warburg effect, as well as increased ammonia recycling, glutamate metabolism and purine metabolism (Figure 3A and Table 2). Relative to non-aspirin users that did not respond favorably to B vitamin supplementation, responders also displayed enhanced spermidine and spermine biogenesis (Figure 3B). Comparing with subjects on B vitamin supplementation with concurrent aspirin usage, subjects not on aspirins also exhibited marginally enhanced propanoate metabolism (Figure 3C). Concurrent aspirin usage largely abrogated the enhanced glucose metabolism brought about by B vitamin supplementation, and no metabolic pathways were significantly altered with respect to the placebo + aspirin control group (Figure 3D).

**TABLE 2:** Summary of top altered pathways.

Comparisons	Metabolite set	Total	Expected	Hits	Fold	Raw *p*	Direction
Active non-aspirin CDR-SOB decreased/Placebo non-aspirin	Gluconeogenesis	2	0.204	2	9.8	0.00947	Upregulated
	Warburg effect	6	0.612	3	4.9	0.0133	Upregulated
	Ammonia recycling	8	0.816	3	3.68	0.0332	Upregulated
	Glutamate metabolism	8	0.816	3	3.68	0.0332	Upregulated
	Purine metabolism	10	1.02	3	2.94	0.0633	Upregulated
	Arginine and proline Metabolism	11	1.12	3	2.68	0.082	Upregulated
Active non-aspirin CDR-SOB decreased/active non-aspirin CDR-SOB unchanged	Spermidine and spermine biosynthesis	4	0.0408	1	24.51	0.0408	Upregulated
	Urea cycle	9	0.0918	1	10.89	0.0918	Upregulated
Active non-aspirin CDR-SOB decreased/active aspirin	Propanoate metabolism	4	0.0816	1	12.25	0.0804	Upregulated
	Valine, leucine and Isoleucine Degradation	5	0.102	1	9.8	0.0999	Upregulated
Active aspirin/placebo aspirin	Phenylalanine and tyrosine metabolism	5	0.102	1	9.8	0.0999	Downregulated

### Metabolism Underlying Inter-Individual Variability to B Vitamin Supplementation

**FIGURE 4:**
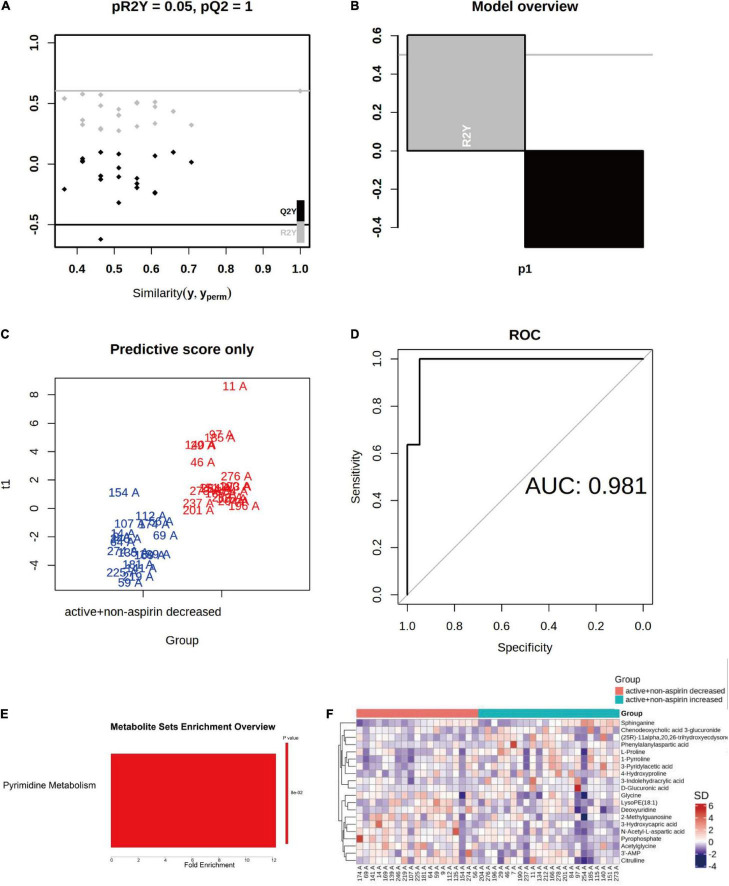
Differences in baseline metabolome profiles between non-aspirin users on active B vitamin supplementation who responded positively (i.e., reduction in CDR-SOB) and negatively (i.e., increase in CDR-SOB) over the intervention period of 24 months. **(A–D)** OPLS-DA analysis of baseline serum metabolomes between the two groups; **(E)** Top altered metabolic pathway between the two groups; **(F)** Heatmap illustrates top altered metabolites based on VIP scores from OPLS-DA analysis.

While it is clear from previous randomized trials that aspirin interacted with B vitamin supplementation to influence the cognitive outcome of the intervention (10, 11), the effects of B vitamin supplementation toward cognitive outcome in non-aspirin users were also varied. To delve into the potential underlying metabolic differences that may have influenced the outcome, we then investigated the differences in baseline metabolome profiles of non-aspirin users who responded positively and negatively, respectively, to B vitamin supplementation over a course of 24-months. The baseline metabolome profiles between non-aspirin users on active B vitamin supplementation experiencing a drop in CDR-SOB were markedly different from those that exhibited increase in CDR-SOB. The baseline metabolome profiles were clearly segregated based on orthogonal projections to latent structures discriminant analysis (OPLS-DA) with an area under curve (AUC) at 0.981. Due to the relatively smaller cohort size, however, the OPLS-DA model exhibited overfitting (pR2Y = 0.05, pQ2 = 1) (Figures 4A–D). MSEA showed that baseline pyrimidine metabolism was marginally enhanced in individuals who responded positively to active B vitamin supplementation, relative to individuals who responded negatively (Figure 4E). The top metabolites contributing to the observed differences arranged based on VIP scores were illustrated in a heatmap (Figure 4F). Accordingly, deoxyuridine, which constitutes part of pyrimidine metabolism, was amongst the top three altered metabolites, and the baseline levels of deoxyuridines were lower in non-aspirin users who responded negatively to active B vitamin supplementation. Thus, this group of individuals who failed to respond favorably to active B vitamin supplementation exhibited abated pyrimidine metabolism and inherently less efficient folate cycle to effectively couple the conversion of homocysteine into methionine to begin with. They may require longer supplementation period at higher dosage, or may be endogenously incompetent in terms of incorporating and assimilating B vitamins due to deficiencies in specific enzymes. Indeed, a previous work had shown that B vitamin supplementation is effective only in individuals with intact dihydrofolate reductase (17).

## Discussion

Metabolomics analysis revealed a favorable serum metabolome profile in non-aspirin users who responded positively to active B vitamins supplementation, as illustrated by the increases in serum hippuric acid and creatine that contribute positively to cognitive outcome (18, 19). Hippuric acid is a frailty marker and diminished blood concentrations of hippuric acid were reported in elderly Japanese patients with mobility impairment (18). Plasma hippuric acid was also decreased in older patients at high-risk of sarcopenia (20), and dietary supplementation of hippuric acid in the form of blueberries led to cognitive enhancement and improved performance in verbal learning test (21). On another note, oral creatine supplementation promotes corticomotor excitability and cognitive performance during acute oxygen deprivation in adults (22), and is associated with improvements in short-term memory and reasoning of healthy individuals (19). In addition, while active B vitamin supplementation increased serum THA-G, while concurrent aspirin usage abrogated this increase. Aldosterone is metabolized to dihydro- and tetrahydro derivatives that subsequently undergo glucuronidation in the liver, with THA-G accounting for majority of daily aldosterone elimination *via* the urine (23). Given the role of aldosterone as a relevant factor in the evolution of arterial hypertension (24), impeded glucuronidation and the subsequent elimination of serum aldosterone in the form of THA-G may be detrimental toward vascular health. The current metabolomics profiles suggest that active B vitamin supplementation is associated with enhanced glucuronidation of aldosterone. Thus, the general serum metabolome profiles of active + non-aspirin group who responded positively was associated with enhanced levels of metabolites positively associated with cognitive improvement and vascular health. Concurrent aspirin usage largely abrogated these potentially beneficial metabolite changes associated with active B vitamin supplementation.

Enhanced gluconeogenesis and glycolysis revealed by MSEA analyses in non-aspirin responders are expected to contribute positively toward cognitive maintenance. Drastic reductions in cerebral glucose metabolism and utilization are common patho-biochemical features of dementia of the Alzheimer type (25, 26). Restoration of glucose metabolism salvages amino acids and lipids that would otherwise be expended as fuel reserves when cerebral glucose metabolism falls below critical thresholds, thereby preserving critical lipid membranes and proteins crucial to maintaining normal brain function. Enhanced glucose metabolism also counteracts increases in glycated hemoglobin and attenuates the accumulation of advanced glycation end-products, which contributes to memory improvement in mice (27). Interestingly, enhanced Warburg effect was also observed in non-aspirin responders. Warburg effect, first observed by Otto Warburg in cancer cells, refers to the preferential dependency on glucose utilization, marked by elevated glycolysis and lactate production regardless of oxygen bioavailability. Biochemical measurement in port-mortem brain tissues of Alzheimer’s disease (AD) patients, which denotes an assessment of the biochemical activities of viable beta-amyloid (Aβ)-resistant cells instead of susceptible, dying cells (28, 29), uncovered an elevation of glycolytic enzymes in the AD brains relative to that of age-matched controls. In addition, clonal nerve cell lines and primary cortical neuron resistant to Aβ toxicity exhibited elevated flux of glucose through the glycolytic pathway, attributed to the activation of the transcription factor hypoxia inducible factor 1 (HIF-1) (29). Furthermore, the existence of a specific group of asymptomatic individuals without pathological history of dementia but having high accumulation of Aβ plaques adds to the hypothesis that altered cellular metabolism, particularly enhanced glucose utilization *via* glycolysis, may offer resistance against the neurotoxic effects of Aβ (30). Based on these observations, it was proposed that neurons may acquire resistance to Aβ accumulation at the incipient stages of AD by skewing cellular metabolism toward enhanced glycolysis and lactate generation, which serves to silence mitochondrial oxidative phosphorylation, reducing the generation of reactive oxygen species that trigger apoptosis and neuronal death (31). These metabolic adaptations akin to Warburg effects preserve neuronal integrity and protect cognitive functions. Thus, active B vitamin supplementation in a specific group of non-aspirin users triggered enhanced Warburg effect that may contribute positively toward neuron survival in MCI individuals.

Non-aspirin users who responded favorably to active B vitamin supplementation also exhibited elevated purine metabolism, ammonia recycling and glutamate metabolism. Enhanced purine metabolism is likely attributed to the increased flow of metabolites through the folate cycle as a result of active B vitamin supplementation (32). Elevated ammonia recycling and glutamate metabolism may arise from the enhanced glutamine-glutamate cycle mediated by glutamine synthetase (GS). In the brain, GS serves to recover glutamate released by neurons by converting them to glutamine in astrocytes, which are then transported to neurons for subsequent conversion to glutamate again. This cycle replenishes the excitatory neurotransmitter glutamate essential for maintaining normal cognitive functions. Accordingly, GS expression was reduced in senile dementia of the Alzheimer type (33). The purine nucleotide cycle liberates ammonia (34), which the brain detoxifies by binding to alpha-ketoglutarate and glutamate to produce glutamine (35). Excess ammonia is detrimental to astrocytes and may lead to gliosis observed in early neurodegeneration in dementia (36). Indeed, AD brains were found to release a larger amount of ammonia (36). Enhanced ammonia recycling *via* glutamine formation and elevated purine metabolism in non-aspirin users who responded positively to active B vitamins thus serve to maintain astrocyte-neuron coupling pivotal to normal brain function.

The cognitively favorable changes were not observed in subjects who took aspirin concurrently, and a marginal difference in propanoate metabolism may partly explain the differing cognitive outcome. Compared to non-aspirin users, propanoate metabolism was downregulated in subjects on active B vitamin supplementation with concurrent aspirin usage. Propionate is a gut microbe-derived metabolite that positively modulates the peripheral and central nervous system. In particular, levels of fecal propionate were negatively correlated with Aβ-42 levels in MCI subjects (37). Based on our metabolomics analysis, aspirin may interact with active B vitamins in a manner that is mediated by gut microbial metabolism. Indeed, vitamin B12 modulates gut microbial ecology and influences host-microbe symbiosis in humans (38). The gut microbial metagenome displays considerable interpersonal variations, more so than that observed in host gene expressions, and may represent an important factor in determining inter-individual differences in disease predisposition and responses to treatment (38). Spermine and spermidine metabolism was enhanced in non-aspirin users who displayed improvement in CDR-SOB on active B vitamin supplementation compared to individuals who did not. The biosynthesis of major polyamines including spermidine and spermine from putrescine is coupled to the conversion of S-adenosylmethionine into decarboxylated S-adenosylmethionine (39), and is henceforth affected by the flux through the folate cycle (40). Gut microbiota is responsible for production of bulk polyamines in the lower part of the intestine (39), and spermines and spermidines were shown to delay brain ageing by promoting autophagy and improving mitochondria function (41). In non-aspirin users who did not respond well to active B vitamins supplementation, it is possible that their gut microbial configurations hindered effective vitamin B12 uptake. Indeed, subjects with high bacterial loads in their small intestines were found to possess low vitamin B12 (42), and specific gut microbe populations may compete with host for dietary vitamin B12 bioavailability in the small intestines (38).

This study has limitations. While our metabolomics analysis herein put forth enhanced Warburg effect and preferential glucose utilization as possible contributors to cognitive improvement brought about by active B vitamin supplementation in non-aspirin users, the current study design does not allow the interpretation of causality. We cannot conclude that these metabolic pathways alterations were resulted from active B vitamin supplementation alone, amidst other confounding factors such as dietary preferences that were not monitored; or that these pathway alterations actually lead to cognitive improvement reflected by the reduction in CDR-SOB. Furthermore, our study makes the basic assumption that systems metabolic alterations in the serum are reflective of changes in neuro-metabolism. Further investigation in larger cohort of subjects from different ethnicities, and preferably based on cerebrospinal fluid samples, may allow a closer reflection of neuro-metabolic adaptations in response to active B vitamin supplementation. To determine causality between B vitamins supplementation and cognitive improvement mediated by enhanced Warburg effects and glucose utilization in brain cells, mechanistic studies using cell cultures and animal models are imperative. Finally, it may be worthy to examine the gut microbial compositional changes in fecal samples of future human cohorts subjected to active B vitamin supplementation, to determine if distinct microbial communities may underlie inter-individual differences in terms of their responses to B vitamin intervention observed in our study.

## Data Availability Statement

The original contributions presented in the study are included in the article/Supplementary Material, further inquiries can be directed to the corresponding authors.

## Ethics Statement

The trial was approved by the Medical Ethics Committee of Chinese University of Hong Kong and Hospital Authority of Hong Kong (CUHK_CCT00373). The patients/participants provided their written informed consent to participate in this study.

## Author Contributions

TK: conceptualization and project administration. HT and SL: methodology. BJ and BL: formal analysis and visualization. HZ, YW, and ML: investigation. SL: writing—original draft preparation. TK, YW, BL, GS, and HZ: writing—review and editing. SL and TK: supervision. TK and GS: funding acquisition. All authors have read and agreed to the published version of the manuscript.

## Conflict of Interest

HZ BJ, BL, and SL are employees of LipidALL Technologies. The remaining authors declare that the research was conducted in the absence of any commercial or financial relationships that could be construed as a potential conflict of interest.

## Publisher’s Note

All claims expressed in this article are solely those of the authors and do not necessarily represent those of their affiliated organizations, or those of the publisher, the editors and the reviewers. Any product that may be evaluated in this article, or claim that may be made by its manufacturer, is not guaranteed or endorsed by the publisher.

## Supplementary Material

Supplementary Table 1 Clinical information.

Supplementary Table 2 Metabolome dataset.
